# Case Report: Trigeminocardiac Reflex in Endovascular Recanalization of Intracranial Internal Carotid Artery Occlusion

**DOI:** 10.3389/fneur.2022.902620

**Published:** 2022-07-13

**Authors:** Hecheng Ren, Yubo Wang, Bin Luo, Lin Ma, Yuxiang Ma, Long Yin, Ying Huang

**Affiliations:** Department of Neurosurgery, Tianjin Huanhu Hospital, Tianjin, China

**Keywords:** trigeminocardiac reflex, internal carotid artery occlusion, interventional complications, endovascular recanalization, ischemic stroke

## Abstract

**Background:**

The trigeminocardiac reflex (TCR) is a unique brainstem reflex that manifests as sudden negative hemodynamic changes. Although rare, TCR may develop during interventional neuroradiology procedures. Intracranial internal carotid artery occlusion (ICAO) is a cause or risk factor of ischemic stroke. Endovascular recanalization is an effective treatment for intracranial ICAO. The occurrence of TCR during the endovascular treatment of intracranial ICAO has not been reported previously.

**Methods:**

We identified and reviewed four intracranial ICAO cases who suffered a sudden negative hemodynamic change during endovascular therapy at our hospital between March 2019 and December 2020.

**Results:**

There were five sudden heart rate and/or blood pressure drops in the four cases; all occurred just after contrast agents were injected. Some angioarchitectural characteristics were common among the four cases. First, the intracranial internal carotid artery distal to the ophthalmic artery was occluded, leaving the ophthalmic artery as the only outflow tract. Second, there were obstructive factors proximal to the end of the guiding catheter, including a vasospasm or dilated balloon. This type of angioarchitecture with a limited outflow tract creates a “blind alley.” The five negative hemodynamic events all recovered: two spontaneously and three after drug administration. Postoperatively, two of the four patients developed ocular symptoms.

**Conclusions:**

Intracranial ICAOs may produce a distinctive angioarchitecture, such as a blind alley, that predisposes patients to TCR. Surgeons should pay special attention to the possibility of TCR during the endovascular recanalization of intracranial ICAO. Low-pressure contrast injections should be attempted, and anticholinergics should be ready for use.

## Introduction

The trigeminocardiac reflex (TCR) is a unique brainstem reflex that manifests as sudden negative hemodynamic changes, including a sudden lowering of both the heart rate and mean arterial blood pressure, cardiac arrhythmias, asystole, and other autonomic reactions such as apnea and gastric hypermotility (1). TCR is thought to be associated with the stimulation of sensory branches of the trigeminal nerve. The hemodynamic changes can often return after a stimulus has ceased, and the inadequate awareness or inappropriate treatment of TCR can lead to catastrophic consequences. TCR may occur in a variety of diseases as well as during or after surgery involving the trigeminal nerve (most commonly neurosurgery or ophthalmic surgery). Although rare, TCR can also be encountered during interventional neuroradiology procedures (2–5).

Intracranial internal carotid artery occlusion (ICAO) is a cause or risk factor of ischemic stroke (6). Endovascular therapy for selected patients with acute ICAO has definite benefits and has been widely accepted. Endovascular recanalization of non-acute ICAO has also been selectively performed, with promising results (6). Here, we report four cases of intracranial ICAO with sudden negative hemodynamic changes during endovascular therapy, which were probably TCRs. We suggest that intracranial ICAOs have distinctive angioarchitectural characteristics that are prone to TCR, and that particular attention should be paid to TCR in the endovascular recanalization of intracranial ICAOs.

## Methods

We identified and reviewed four patients with intracranial ICAO who suffered a sudden negative hemodynamic change during endovascular therapy at our hospital between March 2019 and December 2020. Ethical approval was obtained from the Human Research Ethics Committee of our hospital and written consent was obtained from all patients.

## Results

### Case 1

**Figure 1:**
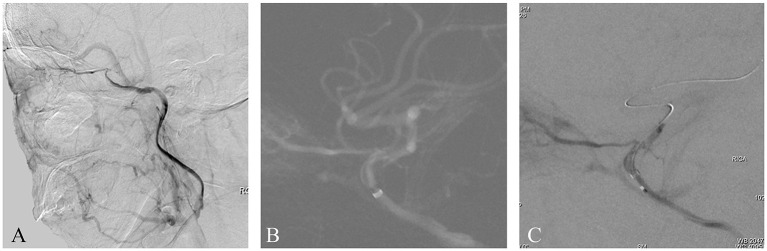
**(A)** This is a non-acute ICAO. The intracranial ICA was occluded, and the ophthalmic artery was the only outflow tract. **(B)** A Navien 6F catheter was placed as close as possible to the occluded segment. A roadmap was generated by manual injection, whereupon the patient's heart rate and blood pressure suddenly dropped. **(C)** After a microwire and microcatheter were passed through the occluded site, another manual injection was performed, whereupon another serious drop in heart rate and blood pressure occurred. Finally, the operation was aborted.

A patient presented with transient weakness in both left limbs in the preceding 2 weeks. Six months previously, the patient suffered a stroke resulting in left limb weakness, which recovered after medical treatment. The patient had a 2-year history of hypertension but no history of diabetes or heart disease. Magnetic resonance imaging (MRI) showed some non-acute infarcts in the right basal ganglia and frontal lobe. Computed tomography angiography (CTA) showed an intracranial ICAO, which was confirmed by femoral digital subtraction angiography (DSA) with iohexol contrast agent (Figure 1A). Perfusion-weighted imaging (PWI) revealed hypoperfusion in the occluded intracranial carotid artery (ICA) territory. There were no obvious contraindications on preoperative examination. Endovascular treatment was performed using iohexol as a contrast agent. After general anesthesia, the patient's heart rate was maintained at ~70 beats per minute (BPM) and their blood pressure around 130/90 mmHg. A Navien 6F, 115 cm, intermediate catheter (Medtronic; Irvine, CA, USA) was placed into the cavernous segment of the right ICA with the support of a 6F, 90 cm, guiding sheath. A roadmap was generated by manual injection through the Navien, whereupon the patient's heart rate suddenly dropped to 14 BPM (Figure 1B). After ~8 s, the heart rate reverted to 55 BPM without medication and the blood pressure was 95/70 mmHg. The operation then continued. A 0.014-inch microwire and a 0.021-inch microcatheter were passed through the occluded site. Another manual injection was then performed, whereupon asystole suddenly occurred and the systolic pressure subsequently dropped to 30 mmHg (Figure 1C). After cardiac resuscitation for 5 min, including chest compressions and atropine, heart rate and blood pressure returned to normal; however, for fear of a catastrophic cardiac complication, the operation was aborted. Postoperatively, the patient suffered decreased vision in the right eye. No other neurological complications occurred and cardiac examinations revealed no cardiac abnormalities. The patient was transferred to an ophthalmic hospital for further treatment. We were unable to obtain the relevant ophthalmic examination data from that hospital, and follow-up data are not available for this patient.

### Case 2

**Figure 2:**
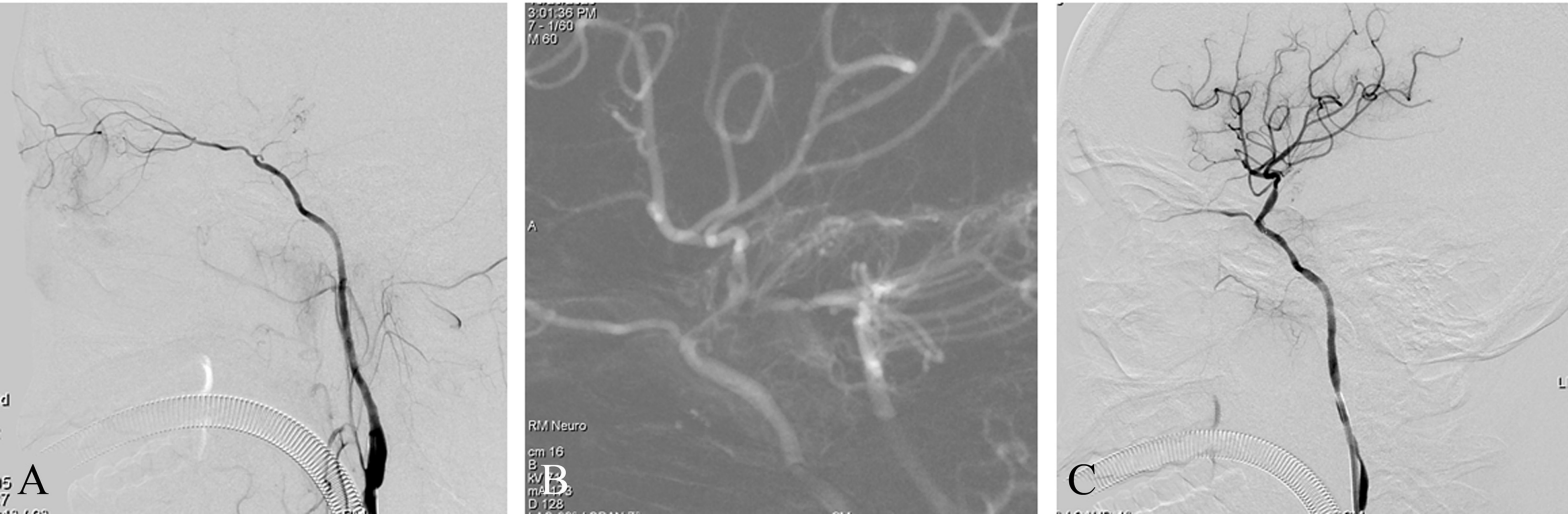
**(A)** This is a non-acute ICAO. The intracranial ICA was occluded, and the ophthalmic artery was the only outflow tract. **(B)** A dual-roadmap was generated manually, whereupon a sudden drop in heart rate occurred, accompanied by a decrease in blood pressure. **(C)** Postoperative angiography showed severe vasospasm of the proximal ICA.

A patient presented with right limb weakness that had suddenly appeared 2 months previously. Imaging studies at onset indicated a watershed cerebral infarction and severe stenosis of the intracranial ICA. The patient's symptoms greatly improved after the initial event, but had worsened over the preceding week. The patient had experienced hypertension for 5 years but no diabetes or heart disease. PWI revealed hypoperfusion in the right middle cerebral artery territory. DSA with the contrast agent iopromide showed an intracranial ICAO (Figure 2A). Endovascular treatment was performed using iopromide. After general anesthesia, the patient's heart rate was maintained at ~60 BPM and the blood pressure at ~115/85 mmHg. A 6F guide catheter was placed into the petrous segment of the ICA and a diagnostic catheter was inserted into the left vertebral artery. A dual vascular roadmap was generated manually by injecting from both the ICA and the vertebral artery (7), whereupon a sudden drop in heart rate occurred (to 30 BPM), accompanied by a decrease in blood pressure to 65/40 mmHg (Figure 2B). After 2 min of intravenous administration of 2 mg atropine, the heart rate and blood pressure returned to normal. The operation then continued and the occluded ICA was successfully recanalized by the passage of a microwire/microcatheter, balloon dilatation, and stent release (Figure 2C). Postoperatively, the patient complained of right ocular and orbital pain, which completely resolved after 3 days. The patient had no visual impairment or other neurological dysfunction.

### Case 3

**Figure 3:**
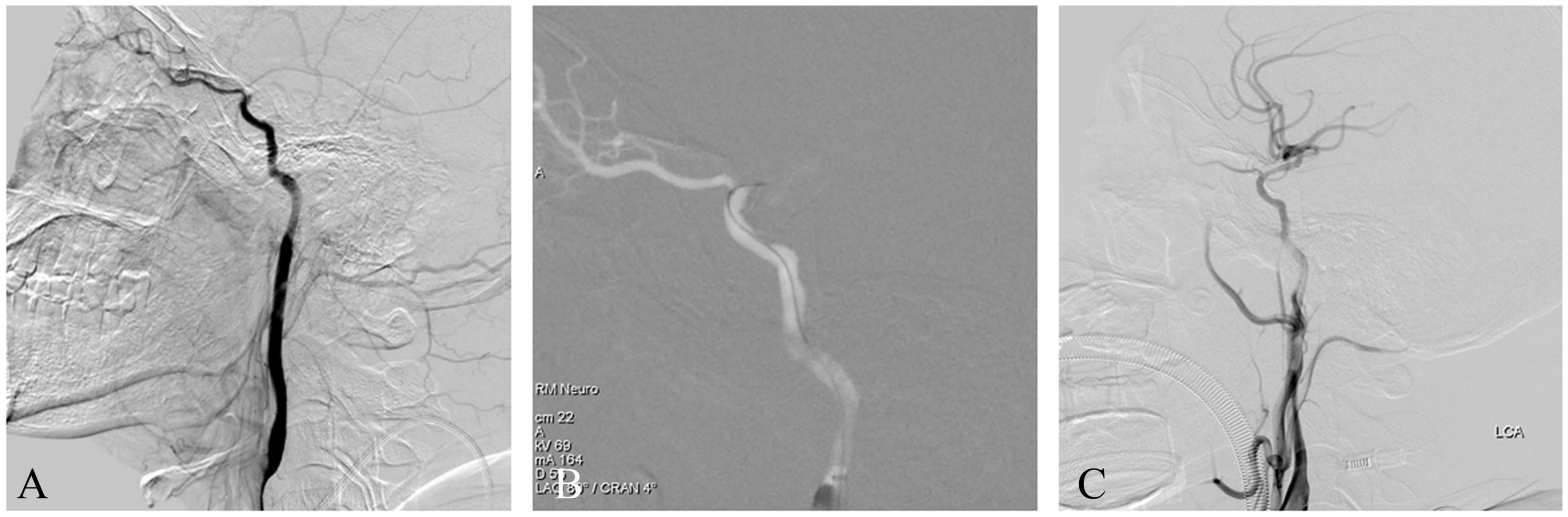
**(A)** This is an acute ICAO. The intracranial ICA was occluded, and the ophthalmic artery was the only outflow tract. **(B)** A balloon guide catheter was used. After the balloon was dilated, a roadmap was manually generated, whereupon the patient's heart rate suddenly dropped, although the blood pressure did not change substantially. The heart rate returned to normal after atropine administration and the operation was continued. **(C)** Postoperative angiography showed that the occluded ICA was recanalized successfully.

This patient suffered an acute ischemic stroke. The patient was admitted having experienced speech impairment and right limb weakness for 11 h, with an NIHSS score of 12. He had a 6-year history of hypertension and a 4-year history of diabetes. Imaging studies indicated a left ICAO and an ipsilateral acute watershed infarction with a large penumbra. Emergent endovascular treatment was performed and a left ICAO was confirmed by diagnostic angiography with iodixanol contrast agent (Figure 3A). After general anesthesia, the patient's heart rate and blood pressure were normal. A balloon guide catheter was placed into the petrous segment of the ICA. A vascular roadmap was then manually generated, whereupon the patient's heart rate suddenly dropped to 40 BPM, although the blood pressure did not change substantially (Figure 3B). Two milligrams of atropine was administered intravenously and the heart rate returned to normal in 1 min. The operation continued routinely, including a stent-retriever thrombectomy and rescue therapy with both balloon dilation and stent release. Finally, the occluded ICA was successfully recanalized and no negative hemodynamic changes recurred (Figure 3C). The postoperative course was uneventful.

### Case 4

**Figure 4:**
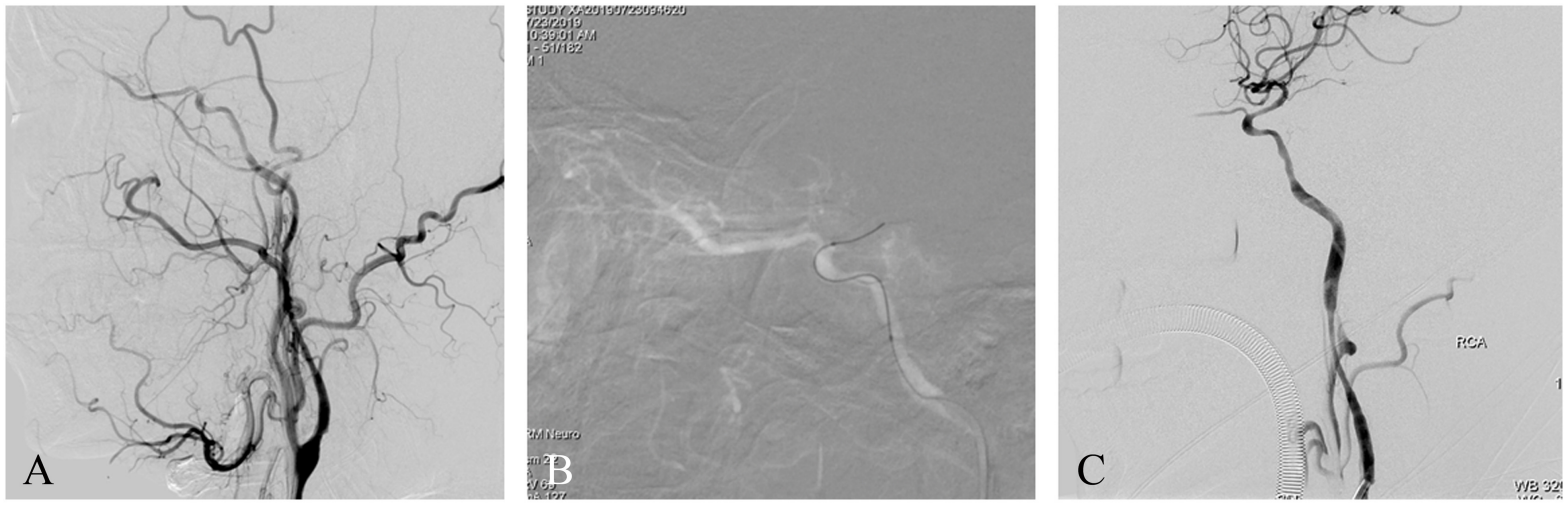
**(A)** This is a non-acute ICAO. The intracranial ICA was occluded, and the ophthalmic artery was the only outflow tract. **(B)** A roadmap was generated manually, whereupon a sudden drop in heart rate and blood pressure occurred. After 1 min, the heart rate and blood pressure recovered spontaneously. The operation was then continued. **(C)** Postoperative angiography showed severe vasospasm of the proximal ICA.

A patient presented with frequent transient left limb weaknesses lasting 1 month. MRI showed some non-acute infarcts in the right watershed area; PWI revealed hypoperfusion in the left middle cerebral artery territory. DSA with the contrast agent iopromide showed an intracranial ICAO (Figure 4A). Endovascular treatment was performed using iopromide. After general anesthesia, the patient's heart rate was maintained at ~70 BPM and the blood pressure at ~130/90 mmHg. A 6F guide catheter was placed into the petrous segment of the ICA. A vascular roadmap was generated manually, whereupon a sudden decrease in heart rate occurred (to 45 BPM), accompanied by a decrease in blood pressure to 90/40 mmHg (Figure 4B). After 1 min, the heart rate and blood pressure recovered spontaneously. The operation was then continued, and the occluded ICA was successfully recanalized by the passage of a microwire/microcatheter, balloon dilatation, and stent release (Figure 4C). The postoperative course was uneventful.

## Discussion

Although iatrogenically induced TCRs are common in neurosurgery and ophthalmology operations, they can also be encountered in interventional neuroradiology procedures. In the field of interventional neuroradiology, TCRs are mainly reported with embolization procedures for arteriovenous fistulas or nasopharyngeal tumors—especially those involving the internal maxillary artery, middle meningeal artery, or cavernous sinus (3–5, 8–10). To the best of our knowledge, a sudden drop in heart rate and/or blood pressure during the endovascular recanalization of ICAO, as in our cases, has not been reported previously.

There were five sudden heart rate and/or blood pressure drops in our four cases. They all happened just after the contrast agents were injected and there were no other possible inducements. We therefore consider that these events were TCRs. TCRs can be induced by a wide variety of mechanical or chemical stimulations of the sensory branches of the trigeminal nerve. Most TCRs in interventional neuroradiology are thought to be related to the toxicity of intravascularly injected drugs, such as dimethyl sulfoxide, Onyx liquid embolic agents, chemotherapeutic drugs, or contrast agents (3–5, 8–10). In the four cases reported here, three different contrast agents were used, and we therefore cannot conclude that they were responsible for the TCRs. TCR can also be caused by high pressure caused by the manual injection of contrast agents into selected arteries, which can stimulate branches of the trigeminal nerve (2). Some of the angioarchitectural characteristics were similar among the four cases. First, the intracranial ICA distal to the ophthalmic artery was occluded. Second, there were obstructive factors proximal to the end of the guiding catheter, including a vasospasm or dilated balloon. Thus, when injection was performed through the guiding catheter, the ophthalmic artery was the only outflow tract and the pressure in the artery and ocular structures would have suddenly risen beyond the normal tolerable range. This would have stimulated the ophthalmic branch of the trigeminal nerve, triggering an oculocardiac reflex, which is a peripheral variant of the TCR and a dysrhythmic physiological response to physical stimulation of the eye. This kind of angioarchitecture, with a limited outflow tract, can be referred to as a “blind alley.” An enormous elevation of intraluminal pressure in the outflow artery by the injection of contrast has been shown by Sadayoshi and colleagues using a silicon vascular model (11). We therefore suggest that high pressure from the contrast injection was the factor that induced TCRs in our four cases. We believe that if the catheter is located in the extracranial carotid or more proximally in the cervical region, the aforementioned reflex will not occur because the contrast agent can reflux into the common carotid artery. Indeed, TCR did not occur during the process of diagnostic angiography in any of these patients. Patients with chronic intracranial ICAOs often experience natural atrophy of the proximal ICA. The narrowed vessel is prone to spasm during catheter placement, which leads to an occlusion proximal to the guiding catheter and a subsequent blind alley. The same mechanism applies to intracranial ICAOs with cervical ICA stenosis or the use of a balloon catheter. Intracranial ICAOs may therefore have a distinctive angioarchitecture that is prone to blind alleys and TCR. Thus, surgeons must be alert to the existence of a blind alley and not use too much pressure when injecting the contrast medium. For some special ICAOs, such as those with preoperative bradycardia, prophylactic medication may be considered.

In addition to TCR, the blind alley angioarchitecture and injection-induced high pressure in the ophthalmic artery may also damage ocular structures and visual function. Glaucoma can arise as a complication of superselective ophthalmic angiography (12). Two of our four patients developed ocular symptoms. One patient suffered severely decreased vision and another had ocular and orbital pain with no visual impairment. These symptoms were probably related to injection-induced high intraocular pressure and/or orbital pressure, although the specific mechanism for these effects is not clear.

In most TCRs, when the stimulus stops, the patient's heart rate and blood pressure improve spontaneously. However, in some cases, medical intervention is necessary. The preferred drugs are anticholinergics, such as atropine, which is effective for most patients (4). However, if the stimulation is intense, the TCR may be refractory to this treatment modality. For some cases with severe bradycardia, adrenaline and a transcutaneous pacemaker may even be used. In three of the five sudden heart rate or blood pressure drops presented here, atropine was used, and all three negative hemodynamic changes returned to normal. We believe that both drug administration and stimulus cessation may help to prevent injection-/pressure-induced TCR in intracranial ICAO surgery.

## Conclusions

These four cases highlight some important considerations. Intracranial ICAOs may have a distinctive angioarchitecture, such as a blind alley, which is prone to TCR. We suggest that surgeons pay special attention to TCR during the endovascular recanalization of intracranial ICAO. Low-pressure contrast injections should be attempted, and anticholinergics such as atropine should be ready for use. We recommend the gentle manual injection of contrast material during interventional procedures for patients with intracranial ICAO. For those with preoperative bradycardia, prophylactic medication may be considered.

## Data Availability Statement

The original contributions presented in the study are included in the article/supplementary material, further inquiries can be directed to the corresponding author/s.

## Ethics Statement

The studies involving human participants were reviewed and approved by Tianjin University Huanhu Hospital. Written informed consent for participation was not required for this study in accordance with the national legislation and the institutional requirements.

## Author Contributions

HR, YW, and YH: study concept and design. HR, YW, and BL: data acquisition and operation. HR and YW: manuscript drafting. HR and YH: review and editing. YH: supervision. All authors contributed to the article and approved the submitted version.

## Conflict of Interest

The authors declare that the research was conducted in the absence of any commercial or financial relationships that could be construed as a potential conflict of interest.

## Publisher's Note

All claims expressed in this article are solely those of the authors and do not necessarily represent those of their affiliated organizations, or those of the publisher, the editors and the reviewers. Any product that may be evaluated in this article, or claim that may be made by its manufacturer, is not guaranteed or endorsed by the publisher.
